# Effects of Consumption of Rooibos (*Aspalathus linearis*) and a Rooibos-Derived Commercial Supplement on Hepatic Tissue Injury by *tert*-Butyl Hydroperoxide in Wistar Rats

**DOI:** 10.1155/2014/716832

**Published:** 2014-03-11

**Authors:** B. D. Canda, O. O. Oguntibeju, J. L. Marnewick

**Affiliations:** Oxidative Stress Research Centre, Department of Biomedical Sciences, Faculty of Health and Wellness Sciences, Cape Peninsula University of Technology, P.O. Box 1906, Bellville 7535, South Africa

## Abstract

This study investigated the antioxidative effect of rooibos herbal tea and a rooibos-derived commercial supplement on *tert*-butyl hydroperoxide- (*t*-BHP-) induced oxidative stress in the liver. Forty male Wistar rats consumed fermented rooibos, unfermented rooibos, a rooibos-derived commercial supplement, or water for 10 weeks, while oxidative stress was induced during the last 2 weeks via intraperitoneal injection of 30 µmole of *t*-BHP per 100 g body weight. None of the beverages impaired the body weight gain of the respective animals. Rats consuming the rooibos-derived commercial supplement had the highest (*P* < 0.05) daily total polyphenol intake (169 mg/day) followed by rats consuming the unfermented rooibos (93.4 mg/day) and fermented rooibos (73.1 mg/day). Intake of both the derived supplement and unfermented rooibos restored the *t*-BHP-induced reduction and increased (*P* < 0.05) the antioxidant capacity status of the liver, while not impacting on lipid peroxidation. The rooibos herbal tea did not affect the hepatic antioxidant enzymes, except fermented rooibos that caused a decrease (*P* < 0.05) in superoxide dismutase activity. This study confirms rooibos herbal tea as good dietary antioxidant sources and, in conjunction with its many other components, offers a significantly enhanced antioxidant status of the liver in an induced oxidative stress situation.

## 1. Introduction 

There has been a steady increase in the popularity of polyphenols/flavonoids and other dietary products as health and wellness promoting agents against excess of free radicals. Epidemiological studies have demonstrated an inverse correlation between the consumption of diets rich in flavonoids and decreased cellular damage via direct radical scavenging of reactive species, chelating of metal ions, or challenging enzymatic systems responsible for free radical production [1–3]. Some of these compounds can activate phase II detoxification enzymes and modify physiological and biotransformation reactions, thus enhancing the xenobiotic detoxification process [4]. Much of the information supporting the usefulness of these nutraceuticals to reverse oxidative stress damage was mainly anecdotal, but in recent years, systematic* in vitro*, animal and human studies have greatly enhanced our knowledge regarding the bioavailability, safety, and efficacy of these compounds as well as their underlying mechanisms of action against oxidative stress [5–8].

Hepatic tissue is greatly exposed to oxidative stress due to its important role in the regulation of various physiological processes including metabolism of carbohydrates and fats, synthesis of bile acids and coagulation proteins, and detoxification of endogenous and xenobiotic compounds among others, exposing itself to free radical injury as a result of sustained exposure to numerous potential toxicants. This damage by free radicals may result from direct or indirect attacks on essential biomolecules leading to the development of diseases such as cancer, nonalcoholic fatty liver disease, and many others [9–12].

*Aspalathus linearis*, commonly known as rooibos, is a shrub native to the Cederberg region in the Western Cape Province of South Africa [13, 14]. It is frequently used to make a mild tasting tisane rich in polyphenol antioxidants but with no caffeine and very little tannins; it is also claimed to cure insomnia, allergies, and nervous breakdown as well as improve the appetite [15, 16]. Currently, many of these claims are at various stages of formal substantiation; however, recent scientific endeavors suggest that rooibos may confer various antioxidant-associated health benefits including antimutagenic, anticarcinogenic, anti-inflammatory, and antiviral properties and antiatherosclerotic effects [16–19]. Some of these health benefits were recently confirmed in humans where the consumption of fermented rooibos improved the lipid profile as well as redox status in adults at risk of developing cardiovascular diseases [20].

Previous and recent studies have demonstrated* Aspalathus linearis* as a good source of unique phenolic compounds exhibiting its protective effect in various tissues [10, 21, 22]. To date, no comparative study has been done that includes a rooibos-derived commercial supplement and compares it to the two forms of rooibos herbal tea, fermented and unfermented, to determine their oxidative stress modulatory effect in liver tissue. The rooibos-derived commercial supplement used in the current study contains 175 mg of a 20% aspalathin-rich extract, 500 *μ*g vitamin A, 150 mg vitamin C, 5 mg Vitamin E, and 25 *μ*g selenium that are hoped to confer a synergistic interaction amongst them. Aspalathin is the main polyphenol present in* Aspalathus linearis* and recent studies have shown that the rooibos antioxidative properties are, in part, attributed to the strong ability to quench radicals generated in the water phase, which can also confer high thermal stability against deep frying [17, 18]. Vitamin C is considered a first line of antioxidant defence system and as the major water soluble antioxidant, acting synergistically with *α*-tocopherol by reducing *α*-tocopheroxyl radicals rapidly to regenerate *α*-tocopherol, and possibly inhibits *α*-tocopheroxyl-mediated radical propagation [23, 24]. Vitamin E is the most bioactive of the isomers and has received much attention due to its radical-scavenging capability in the lipophilic phase [24]. This mixture of molecules acting at different phases and levels of the biological defence system may be more efficient than an isolated compound. These claims were supported in some studies. For example, Bakker et al. [25] studied the anti-inflammatory effects of a dietary mix of resveratrol, green tea extract, tomato extract, *α*-tocopherol, vitamin C, and omega-3 polyunsaturated fatty acids in overweight men where plasma levels of adiponectin, anti-inflammatory and antidiabetic hormone secreted by adipocytes, were increased by 7%, while C-reactive protein was unaffected, oxidative stress biomarkers (isoprostanes and uric acid) were decreased, and concurrently endothelial function was improved.

In the current study* tert-*butyl hydroperoxide (*t*-BHP), a short-chain organic hydroperoxide commonly used in models for oxidative injury in various tissues including hepatic tissue, was used as a free radical generator. After metabolic activation, metabolites such as* t-*butoxyl and* t-*methyl radicals may cause permeabilisation of cell membranes, DNA damage, and depletion of glutathione via a variety of mechanisms [26–28].

This study aimed to assess the redox modulation of fermented and unfermented rooibos herbal tea and a rooibos-derived commercial supplement in the liver tissue of* t*-BHP-induced oxidative stress male Wistar rats.

## 2. Material and Methods

### 2.1. Chemicals

All chemicals used were of analytical grade purity. The Folin Ciocalteu reagent (FCR), 4-dimethylaminocinnamaldehyde (DMACA), hydrochloric acid (HCl), methanol (MeOH), ethanol (EtOH), phosphoric acid (H_3_PO_4_), sodium acetate, glacial acetic acid, ferric chloride, disodium hydrogen orthophosphate dehydrate and dihydrogen orthophosphate-1-hydrate, 2-2′-azino-bis(3-ethylbenzothiazoline-6-sulfonic acid (ABTS^•+^), potassium peroxodisulphate, trichloroacetic acid (TCA), 1,1,3,3,-tetraethoxypropane (TEP), chloroform, cyclohexane, malondialdehyde bisdiethyl acetal (MDA), perchloric acid (PCA), thiobarbituric acid, and sulfuric acid were obtained from Merck (SA), while gallic acid, quercetin, ascorbic acid, fluorescein (FI) sodium salt, 2,2′-azobis(2-methylpropionamidine)dihydrochloride (AAPH), Na-Pi, 0.5%, Triton X-100, 30% hydrogen peroxide, potassium dihydrogen orthophosphate (KH_2_PO_4_), sodium phosphate, ethylenediaminetetraacetic acid (EDTA), glutathione reductase, *β*-NADPH, sodium azide, NaH_2_PO_2_, 6-hydroxydopamine, 1.6 mM (6-HD), diethylenetriaminepentaacetic acid (DETAPAC), 5,5′-dithiobis (2-nitrobenzoic acid) (DTNB), 1-methyl-2-vinylpyridinium (M2VP) glutathione reductase (GR), reduced glutathione (GSH), iron chloride hexahydrate (FeCl_3_ 6H_2_O), 2,4,6-tri[2-pyridyl]-s-triazine (TPTZ), and 6-hydroxy-2,5,7,8-tetramethylchroman-2-carboxylic acid (trolox) were obtained from Sigma-Aldrich (SA).

### 2.2. Preparation of Rooibos Beverages and Supplement

Aqueous extracts of the various plant materials, namely, fermented and unfermented rooibos, were freshly prepared by adding the plant material (superior grade) to freshly boiled tap water at a concentration of 2 g/100 mL (concentrations identical to those used in previous studies) [22, 29]. Firstly, the herbal tea was brewed for 30 min at room temperature before filtering (Whatman N 4), cooled, and then served to the experimental animals in their water bottles. Aliquots of the freshly prepared rooibos herbals tea and rooibos-derived commercial supplement were also stored at −20°C for quantitative antioxidant analyses. The rooibos herbal beverages were freshly prepared every second day. Fermented and unfermented rooibos plant material (superior grade) was a generous gift from Rooibos Ltd. (Clanwilliam, South Africa). The rooibos-derived commercial supplement was crushed using a pestle and mortar and prepared at a concentration of 2 g/100 mL. The rooibos-derived commercial supplement was purchased from a local drug store (Cape Town, South Africa).

### 2.3. Experimental Animals and Diet

Following ethical approval (CPUT/HWS-REC 2010/A001) granted by the Faculty of Health and Wellness Sciences Research Ethics Committee, Cape Peninsula University of Technology, forty male Wistar rats obtained from the Animal Unit of the University of Cape Town (South Africa) weighing 120–150 g were randomly allocated to one of the four study groups. Each group, consisting of ten rats each, was maintained using standard care as described by Awoniyi et al. [22]. Briefly, animals were housed in Perspex houses with stainless wire-bottomed cages in a controlled environment of 24-25°C with 12 hours light-dark cycles and 50% humidity for 1 week. The rats had free access to standard rat chow, the various beverages (fermented and unfermented rooibos and the rooibos-derived commercial supplement), and tap water for the control group for 10 weeks as sole source of drinking fluid. No negative control was included in the current study to ascribe model functionability, as the* t*-BHP concentration used in this study was similar to that used in other studies where it was able to generate oxidative stress [28, 30, 31]. Body weight was monitored twice a week till the end of the experiment. Oxidative stress was induced in all animals via an intraperitoneal (i.p.) injection of 30 *μ*mole of* t*-BHP per 100 g body weight in the final two weeks of the study. At the end of the intervention, the animals were sacrificed under pentobarbital anaesthesia (i.p. 0.4 mL/kg body weight). Thereafter, livers were excised, immediately snap-frozen in nitrogen, and then stored at −80°C. Prior to analyses, the livers were weighed and homogenised according to the specific assay requirements.

### 2.4. Assessment of Phenolic Contents

Assessment of the phenolic contents of the rooibos herbal tea and rooibos-derived commercial supplement included measurements of total polyphenols (TPs), flavanols, and flavonols. The total polyphenol content was determined according to the method described by Waterhouse [32]. Briefly, an 800 mg/L gallic acid stock standard solution was used to prepare the standard series of 0, 20, 50, 100, 250, and 500 *μ*g/L using distilled water as the diluent. The Folin working solution was prepared by diluting 1 mL of Folin Ciocalteu reagent (FCR) with 9 mL of distilled water. The reaction mixture in each 96-microplate well consisted of 25 *μ*L of standard or sample, 125 *μ*L FCR, and 100 *μ*L of sodium carbonate (7.5%) added after 5 min and read in a Multiskan Spectrum plate reader (Thermo Electron Corporation, USA) set at 25°C and 765 nm. The polyphenol content was expressed as mg gallic acid equivalents per litre of beverage or supplement (mg GAE/L). The flavanol and flavonol contents of the fermented and unfermented rooibos herbal tea and rooibos-derived commercial supplement were determined colorimetrically at 640 nm and 360 nm, using the methods described by Treutter et al. [33] and Mazza et al. [34], respectively, with flavanol contents expressed as mg catechin equivalents (CE) per litre and flavonol contents expressed as mg quercetin equivalents (QE) per litre.

### 2.5. Estimation of Antioxidant Capacity

The antioxidant capacity of the various rooibos herbal tea and rooibos-derived commercial supplement consumed in the study was determined using the oxygen radical absorbance capacity (ORAC) assay, while that of the rat liver tissue was assessed using three different assays, that is, the ferric reducing antioxidant power (FRAP) assay, the ORAC assay, and the ABTS+ assay expressed as trolox equivalent antioxidant capacity (TEAC). The FRAP assay was used as described by Benzie and Strain [35] to assess the ability of the sample to reduce the 2,4,6-tri[2-pyridyl]-s-triazine (Fe^3+^) to the ferrous (Fe^2+^-TPTZ complex) state at low pH, forming an intense blue-coloured complex with optimal absorbance at 593 nm, while the ABTS+ assay was used as described by Re and coworkers [36]. For the FRAP and ABTS+ assays, 300 mg subsamples of the liver tissue were homogenized in 1.2 mL of freshly prepared 75 mM phosphate buffer (pH 7.0) following centrifugation (Eppendorf 5810R, Eppendorf, Germany) for 30 min at 4000 rpm, 4°C, and used as is. Data analyses were carried out using Microsoft Excel 2003 based on a calibration curve plotted using a standard dilution series of ascorbic acid (AA) and trolox (T), respectively. The ORAC assay, as described by Ou et al. [37], was used to estimate the antioxidant capacity of the various rooibos herbal tea types (required a 20-fold dilution in phosphate buffer) and liver tissues (required proteins to be precipitated with 5% PCA and also a 20-fold dilution in phosphate buffer); each reaction mixture consisted of 12 *μ*L of protein-free sample or standard and 138 *μ*L of fluorescein (FI) and was initiated by the addition of 50 *μ*L of 2,2′-azobis(2-methylpropionamidine)dihydrochloride (AAPH) solution. The activity was determined using the regression equation *y* = *ax* ^2^ + *bx* + *c*. All assays were done in triplicate and results were expressed as *μ*mole AAE/g tissue, *μ*mole TE/g for the tissue samples, and *μ*mole TE/L for the rooibos herbal tea and derived supplement.

### 2.6. Estimation of Lipid Peroxidation Products

Estimation of lipid peroxidation products was done by measuring thiobarbituric acid reactive substances (TBARS), which reflect the production of malondialdehyde (MDA), a product of lipid oxidation. The use of high-performance liquid chromatography (HPLC) with fluorescence detection circumvents some of the limitations of previous methods and was therefore used in the current study. The method described by Recknagel and Glende [38] was used, with the MDA-TBA adduct analysed using HPLC. Briefly, the thawed aliquots (250 *μ*L) of liver tissue homogenates were pipetted into 1.5 mL reaction tubes containing 50 *μ*L, 6 M sodium hydroxide and incubated at 60°C for 30 min for protein hydrolysis. Thereafter, 125 *μ*L of 35% perchloric acid was added to precipitate proteins, after the mixture was centrifuged at 4°C, 2800 rpm for 10 min. Supernatants (100 *μ*L) were pipetted into new reaction tubes containing 250 *μ*L 40 mM TBA and 750 *μ*L 0.2 M orthophosphoric acid. This mixture was boiled at 100°C for 1 hr, cooled on ice, and centrifuged (Eppendorf AG 5810R, Germany) at 14000 rpm for 4 min. The MDA standard was prepared by dissolving 25 *μ*L 1,1,3,3-tetraethoxypropane (TEP) in 100 mL water to provide a 1 mM stock solution. Working standards were prepared by mixing 1 mL TEP stock solution in 50 mL 1% sulphuric acid incubated for 2 hr at room temperature. The resulting MDA standard (20 *μ*mole/L) was further diluted with 1% sulphuric acid to final concentrations of 0.5, 1, 2, 5, and 10 *μ*M for determination of the standard curve for the estimation of MDA. The reaction mixture (20 *μ*L) was injected into an Agilent Technology 1200 Series (Germany) HPLC system for separation of the MDA-TBA complex. The mobile phases used were as follows: A: 100% methanol and B: potassium dihydrogen phosphate (KH_2_PO_4_) buffer (50 mM, 5.8 pH) in a ratio (A : B) 60% : 40%. A flow rate of 0.8 mL/min and fluorescent detection with excitation wavelength set at 532 nm and emission wavelength set at 552 nm were used. Results were expressed as nmole/mg in the tissue extract sample.

### 2.7. Measurement of Antioxidant Enzymes

The three enzymes, namely, catalase (CAT), glutathione peroxidase (GPx), and superoxide dismutase (SOD), were measured to determine liver antioxidant enzymes activity. The tissue samples were prepared by homogenising 300 mg in 2 mL of phosphate buffer followed by centrifugation at 15 000 g, 10 min, 4°C, and subsequent separation of the supernatant. CAT activity was assessed spectrophotometrically by the modified method of Ellerby and Bredesen [39]. GPx activity was determined using the method of Flohé and Günzler [40] and SOD as described by Ellerby and Bredesen [39]. The two assays were done in duplicate and enzyme activity of catalase in the sample was calculated based on the rate of decomposition of hydrogen peroxide which is proportional to the reduction of absorbance at 240 nm wavelength, while glutathione peroxidase activity in the sample was calculated based on the rate of decrease in absorbance at 340 nm wavelength over the time using the molar extinction coefficient of glutathione peroxidase and corrected for path length. The estimation of SOD activities was done in duplicate using a standard curve with SOD at different concentrations. All results were expressed as *μ*mole/min/*μ*g protein.

### 2.8. Protein Determination

Protein concentrations of all liver homogenates and/or supernatants were determined using the Pierce bicinchoninic acid (BCA) protein assay, with bovine serum albumin as standard. Results were expressed as mg protein/mL tissue supernatant of homogenate.

### 2.9. Statistical Analyses

Results were expressed as a means ± standard deviation (S.D) for each group tested. Two- way analysis of variance (ANOVA) complemented with Duncan Bonferroni Alpha for multiple comparison and Spearman's Rank Correlation Coefficient tests were used to compare the groups and assess the strength of their associations. In all analyses, the level of significance was set at *P* < 0.05.

## 3. Results

### 3.1. Antioxidant Profile of the Rooibos Beverages and Supplement

Results of the polyphenol, flavonol, and flavanol contents and oxygen radical absorbance capacity of the rooibos herbal tea and rooibos-derived commercial supplement (DCRS) are shown in Table 1. The DCRS contained significantly (*P* < 0.05) higher levels of total polyphenols, which may reflect the purified and rich phenolic compound present in the mixture, when compared to the fermented and unfermented rooibos herbal tea, while as expected the DCRS also presented the highest antioxidant capacity, as determined by the ORAC assay.

### 3.2. Daily Beverage Intake and Body Weight

Neither the rooibos herbal tea nor rooibos-derived commercial supplement was associated with any changes in body weight gains, Table 2. Rats consuming the derived supplement showed a significantly (*P* < 0.05) lower daily fluid intake, but this did not impair significantly their body weight gains. The highest (*P* < 0.05) daily polyphenol intake was achieved by rats consuming the derived supplement, followed by rats consuming the unfermented rooibos and then the fermented rooibos. When considering their daily ORAC intake, it was the rats consuming the unfermented rooibos that showed the highest (*P* < 0.05) intake, while the ORAC intake was similar (*P* > 0.05) for the fermented rooibos and derived supplement groups. Although the daily fluid intake of rats consuming the fermented and unfermented rooibos was higher (*P* < 0.05), their daily intake of polyphenols was lower (*P* < 0.05) when compared to the daily intake of the derived supplement. It is known that the absorption phase impacts on biodisponibility of individual polyphenol content [41, 42]. Rats consuming the rooibos herbal tea had the highest (*P* < 0.05) flavonol intake when compared to the group consuming the derived supplement.

### 3.3. Hepatic Antioxidant Capacity

The comparative data of the hepatic antioxidant capacity (AOC) in the liver of rats consuming the rooibos herbal tea and rooibos-derived commercial supplement after* t*-BHP-induced oxidative stress are shown in Table 3. With the exception of FRAP activity in all the groups, there was a significant (*P* < 0.05) increase in the hepatic AOC in the rats consuming the unfermented rooibos as well as the derived supplement when compared to the water control and fermented rooibos groups. These increases were significant (*P* < 0.05) when considering ORAC (unfermented rooibos and the derived supplement groups) and ABTS+ (derived supplement group).

### 3.4. Lipid Peroxidation

The effect of the dietary antioxidant interventions on* t*-BHP-induced oxidative damage using MDA as marker of lipid peroxidation is shown in Table 4. None of the rooibos interventions had any significant effect on the* t*-BHP-induced MDA elevation in the liver when compared to the control group consuming water. However, a decreasing trend in this marker was noted in all the groups, with the group consuming the rooibos-derived commercial supplement showing the lowest value (121 nmole/mg ± 22  versus 153 nmole/mg ± 34  for the control).

### 3.5. Hepatic Antioxidant Enzymes

Data on the activities of hepatic antioxidant enzymes of rats consuming the various rooibos herbal tea and rooibos-derived commercial supplement are presented in Table 5. It has been reported that treatment with* t-*BHP significantly reduces glutathione-related enzyme activities [43]. In the current study, when compared to the control group, none of the dietary antioxidant interventions showed any significant changes in the hepatic CAT and GPx activities. When considering the hepatic SOD activity, the group consuming the fermented rooibos showed a significant (*P* < 0.05) decrease in the hepatic activity of this enzyme when compared to the control group consuming water. In the same intervention group, correlation coefficient found that superoxide dismutase was positively correlated with ORAC and TEAC (*r* ^2^ = .661, *P* = 0.038 and *r* ^2^ = .867, *P* = 0.001, resp.).

## 4. Discussion 

There is substantial evidence from human and experimental animal studies that biomarkers of oxidative damage present in plasma, urine, and cells are increased in subjects with risk factors for or displaying certain disease conditions, as well as in certain physiological conditions, such as in intense and prolonged exercise. For example, the* in vivo* markers for lipid peroxidation, F_2_-isoprostanes, and their metabolites are increased in liver and neurological disorders and in individuals with CVD risk factors, such as cigarette smoking, diabetes mellitus, obesity, and hypercholesterolemia [44–46]. Disease developing from oxidative injury to vital biological macromolecules is thus a possibility and the dietary intake of, or supplementation with, antioxidants as a means to reduce the risk for disease development is feasible. However, it is important to note that polyphenols are a heterogenous group of compounds, and their antioxidant properties may be affected by their absorption, distribution, retention, metabolism, and related enzymatic levels leading to the production of putative metabolites with different antioxidant activities [23, 42].

The current study confirms that fermented rooibos herbal tea has a lower total polyphenol content when compared to its unfermented counterpart, as the fermentation process (an oxidative environment) is known to decrease the total polyphenol concentration due to structural and enzymatic changes taking place [16, 47]. The rooibos-derived commercial supplement (RDCS) showed a significantly higher polyphenol content due to the presence of the aspalathin-rich extract. During fermentation, the dihydrochalcone content of rooibos is decreased with less than 7% of the original aspalathin content remaining in fermented rooibos [47]. The presence of this extract as well as the various antioxidant vitamins and trace elements in the rooibos-derived supplement explains its higher antioxidant capacity when compared to the rooibos herbal tea. Antioxidants are known to interact with each other sometimes synergistically through overlapping or complementary effects but this may not be the case in a dynamic and complex biological system where compounds competing for the same functional site may result in inhibition [48–50]. Future studies should be aimed at unravelling these complex interactions to further elucidate the current findings.

The hepatic antioxidant status of the* t*-BHP-induced oxidative stress rats was evaluated using three different antioxidant capacity assays, that is, ORAC, FRAP, and ABTS+, as these assays use different endpoint measurements and quantification approaches [51, 52]. Also, it is important to keep in mind that biological systems are complex and characterised by multiple mechanisms involved in oxidative stress. On the basis of that, previous authors have recommended a battery of assays rather than a single assay approach, keeping in mind the specific research question to be addressed [23, 53–55]. In the current study, none of the intervention groups could significantly influence the hepatic FRAP measurement. However, the intake of unfermented rooibos and the rooibos-derived commercial supplement significantly restored the* t*-BHP-induced reduction of AOC and increased the hepatic antioxidant status with regard to ORAC and TEAC levels. A previous study by Marnewick et al. [18] also showed that unfermented rooibos restored and enhanced the injured livers' ORAC levels in rats exposed to diethylnitrosamine (DEN). In 2010 Villaño et al. [56] showed for the first time that both fermented and unfermented rooibos teas are able to increase plasma antioxidant defences in a human study. ORAC measures the degree of inhibition of peroxyl-radical-induced oxidation by a compound and rooibos's scavenging capacity or antioxidant activity may account for potential effects of secondary products. From the results obtained in this study, it would be difficult to deduce exactly which group of compounds was responsible for this enhanced antioxidant capacity, but it could be speculated that it is due to the polyphenolic compounds, most likely those present at the levels found in the unfermented rooibos. When ingested, dietary flavonoids may cause different benefits as those displayed* in vitro* as a result of absorption, extensive phase III metabolism, and rapid excretion. Results from a previous study confirmed the presence of* O*-linked methyl, sulphate, and glucuronide metabolites of aspalathin in urine of humans that consumed rooibos [57]. These putative metabolites may display different biological activities and could explain, in part, the differences shown in the current study. Unfortunately information regarding the metabolic changes to phenolic compounds present in rooibos herbal tea is scarce and further studies are needed to elucidate this protective effect and the possible role single and/or combinations of compounds pay in the health promoting properties of plant phytochemicals.

The hepatic lipid peroxidation status was assessed using HPLC measuring MDA which has been widely used for determining oxidative stress in mammalian tissues. In this study neither the rooibos herbal tea nor rooibos-derived commercial supplement impacted significantly on the lipid peroxidation status, although a declining trend of TBARS levels in the liver tissues of all intervention groups was noticed. It could be argued that this assay lacks specificity and that the estimates are unlikely to effectively reflect the oxidative injury [58]; however, it is important to note that reports on the effect of polyphenolic intake on lipid peroxidation have demonstrated controversial outcomes. Several studies have suggested a decreased lipid peroxidation level in plasma and hepatic tissue, while others have not [59–61]. Hodgson et al. [62] reported that green tea did not decrease the level of oxidative lipid damage, as measured by urinary F_2_-isoprostanes excretion. Also, the unconjugated flavanol levels in plasma are in the range of 1 *μ*M, which may not be sufficient to yield a greater antioxidant capacity in a complex and dynamic biological system [63]. Future studies should be aimed at elucidating the dose-response relationship of rooibos flavonoids.

As antioxidant enzymes are part of the endogenous defence system and may protect against oxidative stress, none of the rooibos intervention groups showed any further significant increase in the hepatic CAT and GPx activities, when compared to the control group. This supports the findings of Marnewick and coworkers [18] who could not show any increased enzyme activities in liver tissue from rats treated with DEN and consuming the rooibos herbal tea. However, in the present study the hepatic SOD activity in the group consuming the fermented rooibos showed a significant (*P* < 0.05) decrease when compared to the control group consuming water. An increased enzyme activity may be prompted by increased oxidative stress in a negative feedback-like relation, while, conversely, a decreased enzyme activity may reflect a favourable oxidative status balance; that is, enzyme levels decrease as the rate of oxyradicals formation (requiring neutralization) diminishes [64]. In the current study, the positive correlation observed between the hepatic SOD activity and antioxidant capacity (ORAC and TEAC) may strengthen claims that different antioxidants act at different sites as a network to ascribe system efficacy. However, the extent of these correlations among other organs is still to be determined. Similar links between enzymes and antioxidant capacity measures were observed by Dudonné et al. [65] in plants extracts, but in animals models and human studies, correlations between the methods discussed to assess the effect of rooibos herbal tea are not well reported in scientific literature, which suggest the need for further studies that could enhance our current understanding on these matters as a tool for effective study design and interventions.

## 5. Conclusion 

We reported a higher concentration of total polyphenol content in the derived rooibos supplement that may have accounted for the significant higher hepatic oxygen radical antioxidant capacity and the declined trend of lipid peroxidation status in the* t*-BHP-treated rats. Rats consuming the unfermented rooibos herbal tea also showed a significantly enhanced hepatic antioxidant status, while those consuming the fermented rooibos did not. A significant correlation between SOD, ORAC, and TEAC was also found in the livers of rats consuming the fermented rooibos herbal tea. The use of several methodologies to assess the oxidative status displayed results which would otherwise have been missed if only single assays were used. Results from this study suggest that the daily intake of unfermented rooibos herbal tea or a derived commercial rooibos supplement may benefit human health by providing the liver with an enhanced antioxidant capacity to reduce damage induced by toxicants.

## Figures and Tables

**Table 1:** Polyphenol content and antioxidant activity of the rooibos herbal tea and rooibos-derived commercial supplement.

	Aqueous formulations		
	Fermented rooibos	Unfermented rooibos	Rooibos-derived commercial supplement
Total polyphenols (mg GAE/L)	981 ± 118^a^	1354 ± 62^b^	4836 ± 244^c^
Flavanols (mg CE/L)	39 ± 8^a^	92 ± 3^b^	18 ± 10^a^
Flavonols (mg QE/L)	299 ± 49^a^	247 ± 19^b^	181 ± 22^c^
ORAC (µmole TE/L)	14557 ± 905^a^	20889 ± 1281^b^	33768 ± 2322^c^

Values are expressed as mean of three measurements for each aqueous sample ± STD. TE: trolox equivalents; AAE: ascorbic acid equivalents; CE: catechin equivalents; QE: quercetin equivalents; GAE: gallic acid equivalents. Similar letters in rows denote lack of significant difference (*P* > 0.05), but if letters differ then *P* < 0.05.

**Table 2:** Body weight gain and daily fluid intake of *tert*-butyl hydroperoxide-induced oxidative stress rats.

	Supplement groups			
	Fermented rooibos	Unfermented rooibos	Rooibos- derived commercial supplement	Water control
Body weight gain (g)	112 ± 34^a^	135 ± 36^a^	123 ± 36^a^	125 ± 25^a^
Daily fluid intake (mL/day)	74 ± 13^a^	69 ± 15^a^	35 ± 4^b^	69 ± 9^a^
Total polyphenol intake (mg/day)	73.1 ± 8.7^a^	93.4 ± 4.3^b^	169 ± 8.5^c^	ND
Flavanol intake (mg/day)	2.9 ± 0.6^a^	6.3 ± 0.21^b^	0.6 ± 0.35^c^	ND
Flavonol intake (mg/day)	21.8 ± 3.6^a^	17.0 ± 1.3^a^	6.3 ± 0.8^b^	ND
ORAC intake (*μ*mole/day)	1084 ± 67^a^	1441 ± 88^b^	1182 ± 81^a^	ND

Values are expressed as mean ± STD of 10 rats per group. Similar letters in rows denote lack of significant difference (*P* > 0.05), but if letters differ then *P* < 0.05. The daily intake is based on the respective beverage profiles provided in Table 1. ND: not done as these rats consumed tap water. Above intakes were based on fluid intake alone.

**Table 3:** Hepatic antioxidant capacity of rats consuming the rooibos herbal tea and rooibos-derived commercial supplement after *tert*-butyl hydroperoxide-induced oxidative stress.

Antioxidant activity	Supplement groups			
	Fermented rooibos	Unfermented rooibos	Rooibos-derived commercial supplement	Water control
FRAP (µmole AAE/g)	5.36 ± 0.5^a^	4.60 ± 0.5^a^	5.49 ± 2.2^a^	5.01 ± 0.7^a^
ORAC (µmole TE/g)	4.55 ± 1.4^a^	6.53 ± 2.1^b^	6.38 ± 1.2^b^	4.00 ± 2.1^a^
TEAC (µmole TE/g)	41.3 ± 2.0^a^	38.9 ± 2.4^a^	41.9 ± 1.3^b^	37.4 ± 5.4^a^

Values are expressed as mean ± STD of 10 rats per group. Similar letters in rows denote lack of significant difference (*P* > 0.05), but if letters differ then *P* < 0.05. TE: trolox equivalents; AAE: ascorbic acid equivalents.

**Table 4:** Effect of rooibos herbal tea and rooibos-derived commercial supplement on lipid peroxidation in the liver of *tert*-butyl hydroperoxide-induced oxidative stress rats.

Lipid peroxidation levels	Supplement groups			
	Fermented rooibos	Unfermented rooibos	Rooibos-derived commercial supplement	Water control
TBARS (nmole/mg)	135 ± 27^a^	144 ± 21^a^	121 ± 22^a^	153 ± 34^a^

Values are expressed as mean ± STD of 10 rats per group. Similar letters in rows denote lack of significant difference (*P* > 0.05), but if letters differ then *P* < 0.05.

**Table 5:** Hepatic antioxidant enzyme activity in *t*-BHP-induced oxidative stress rats consuming the rooibos herbal tea and rooibos-derived commercial supplement.

Antioxidant activity (µmole/min/µg protein)	Supplement groups			
	Fermented rooibos	Unfermented rooibos	Rooibos-derived commercial supplement	Water control
Catalase	0.153 ± 0.06^a^	0.179 ± 0.04^a^	0.190 ± 0.09^a^	0.261 ± 0.13^a^
Glutathione peroxidase	0.240 ± 0.06^a^	0.233 ± 0.06^a^	0.267 ± 0.05^a^	0.310 ± 0.19^a^
Superoxide dismutase	0.019 ± 0.01^b^	0.028 ± 0.01^a^	0.024 ± 0.02^a^	0.041 ± 0.02^a^

Values are expressed as mean ± STD of 10 rats per group. Similar letters in rows denote lack of significant difference (*P* > 0.05), but if letters differ then *P* < 0.05.
